# Sanwei sandalwood decoction improves function of the gut microbiota in heart failure

**DOI:** 10.3389/fmicb.2023.1236749

**Published:** 2023-10-19

**Authors:** Kuiying Ma, Tingting Bai, Pengfei Hu, Ming Zhao, Zhi Xiu, Surilige, Dalintai, Qingshan Zhang, Quan Wan

**Affiliations:** ^1^Affiliated Hospital of Inner Mongolia University for Nationalities, Tongliao, China; ^2^School of Pharmacy, Jiangxi University of Chinese Medicine, Nangchang, China; ^3^School of Clinical Medicine (Mongolian Medicine), Inner Mongolia University for Nationalities, Tongliao, China

**Keywords:** heart failure, Sanwei sandalwood decoction, intestinal flora, macrogenomics, gut microbiota

## Abstract

**Objective:**

To investigate the effects of Sanwei sandalwood decoction on improving function of the intestinal flora in doxorubicin-induced heart failure in rats.

**Materials and methods:**

Thirty Sprague–Dawley rats were screened and randomly assigned into a blank group, a model group, and a Sanwei sandalwood decoction group (treatment group). The rat model of heart failure was prepared and established in the latter two groups. After successful model establishment, the treatment group received Sanwei sandalwood decoction by continuous gavage at 2 g/kg, once daily for 4 weeks. The other groups were given an equivalent volume of saline. After the final dose, fecal samples were collected from each group and analyzed by macrogenomics and nontargeted metabolomics to characterize the intestinal flora and associated metabolites.

**Results:**

The composition of gut microbiota was significantly different between the three groups. There were 778,808 common genes between the blank and model groups, while 49,315 genes were lost and 521,008 were gained in the model group relative to the blank group. At the phylum level, all groups of rat fecal samples were dominated by *Firmicutes*, *Bacteroidota*, *Actinobacteria*, and *Proteobacteria*. At the genus level, the microbial community composition in all experimental groups of rat fecal samples was dominated by *Lactobacillus*, *Bifidobacterium*, *Limosilactobacillus*, *Allobaculum*, *Prevotella*, and *Ligilactobacillus* spp. Interestingly, cluster analysis was performed on the top 30 KEGG ontology (KO) terms displaying significant differences in relative abundance in the rat fecal microbiome among experimental groups. The relative frequency of posttranslational modification, coenzyme transport and metabolism, cell wall, membrane, and envelope biogenesis in the eggNOG and CAZy databases. In the nontargeted metabolomics, the group principal component analysis revealed that the groups were well distinguished from one another. The different metabolites were screened with VIP >1, and the KEGG different metabolite classification and enrichment analysis revealed that there includes 15 metabolites pathway, including loxoprofen, conifery-l-acetate, trichilin A, and others. The arachidonic acid pathway also accounted for a significant portion of the KEGG pathway classification analysis.

**Conclusion:**

Sanwei sandalwood decoction positively affects the intestinal microbial environment of rats with heart failure, improving the gut dysbiosis that is caused by the condition. This treatment intervention inhibits the growth of pathogenic bacteria and promotes the growth of beneficial species.

## 1. Introduction

Heart failure is a complex clinical syndrome that represents an advanced stage of development of many cardiovascular diseases (Severino et al., 2020). Many morbidities can ultimately lead to heart failure, including cardiomyopathy, valvular heart disease, diabetes, and obesity (Liang et al., 2020; Xu et al., 2020). It affects an estimated 63 million people worldwide, and about 50% of patients die within 5 years of initial diagnosis, identifying heart failure as a global health burden that surpasses most types of cancer as a leading cause of death (Riehle and Bauersachs, 2019). In recent years, it has mainly been treated using the methods of Western medicine, which are associated with significant side effects such as hyponatremia due to administration of diuretics. This often leads to debilitating symptoms such as confusion, falls, and seizures (Lu et al., 2019). Moreover, reduced cardiac output or elevated central venous pressure in heart failure can lead to gastrointestinal dysfunction, localized ischemia, and edema in the gut. In turn, these changes can result in intestinal epithelial dysfunction and barrier defects, flora translocation, and altered composition and metabolites of the intestinal microbiome (Nagatomo and Tang, 2015). In heart failure patients, perturbation of the intestinal flora and their metabolites may therefore be more closely related to development of the condition than for other cardiovascular diseases.

Microbiological studies have traditionally been conducted *in vitro* and with isolated cultures (Sonnenburg and Bäckhed, 2016). However, many gut microorganisms only remain viable in the host and cannot be cultured *ex vivo*, limiting more detailed study of the intestinal flora (Wei et al., 2023). In recent years, contemporary molecular biology techniques such as high-throughput sequencing and real-time quantitative fluorescence polymerase chain reaction (RT-qPCR) (Sender et al., 2016) have been applied for detection and characterization of the intestinal flora. This has enabled the identification of more bacterial species, thus permitting a deeper exploration of the functional spectrum and compositional diversity of the gut microbiome. In this study, the effect of Sanwei sandalwood decoction (i.e., an aqueous extract obtained by boiling) on the intestinal flora investigated in a rat model of heart failure using a macrogenomic approach with the intestinal flora as the entry point. Potential evidence was explored for dysbiosis and altered metabolites in the characteristic gut microflora in heart failure, with the aim of identifying new research directions for treatment of chronic disease.

## 2. Materials and methods

### 2.1. Materials

The experimental protocol and animal use were approved by the Animal Use and Management Ethics Committee at the Animal Experimentation Management Center of Inner Mongolia University of Nationalities (Lot No. NM-LL-2019-10-12-01). The experiment was designed and implemented in accordance with the National Institute of Health Guidelines for the Ethical Use of Animals. All rats were acclimatized for 1 week prior to the start of the study.

### 2.2. Animal experiment

After acclimatization, the rats were assigned to a blank group, model group, or Sanwei sandalwood decoction group (i.e., treatment group) using the random number table method, with 10 rats per group. Animals in the blank group were injected intraperitoneally with saline once a week. To prepare the heart failure model, doxorubicin (saline preparation, 2 mg/mL) was administered intraperitoneally to the remaining rats at a dose of 1.5 mL/kg, once a week for 7 weeks. A combined dose of 21 mg/kg was used to establish the model, in accordance with the literature (Yuhong et al., 2018). After 7 weeks of administration, rats in the treatment group were gavaged with Sanwei sandalwood decoction at a dose of 2 g/kg for 30 consecutive days, while rats in the blank and model groups were given an equivalent volume of saline (Zhou, 2017).

### 2.3. Sample collection and preparation

Rat feces were collected 24 h after the last dose and transferred to lyophilization tubes, then snap frozen in liquid nitrogen and stored in a −80°C freezer (Shao et al., 2020).

The sample stored at −80°C refrigerator was thawed on ice. A 400 μL solution (methanol: water = 7:3, V/V) containing internal standard was added into 20 mg sample, and vortexed for 3 min. The sample was sonicated in an ice bath for 10 min and vortexed for 1 min, and then placed in −20°C for 30 min. The sample was then centrifuged at 12,000 rpm for 10 min (4°C). And the sediment was removed, then centrifuged the supernatant at 12,000 rpm for 3 min (4°C). A 200 μL aliquots of supernatant were transferred for LC-MS analysis.

### 2.4. Preprocessing of sequencing results

The FASTP software was used for raw data quality control. Default software parameters were selected to preprocess the raw data obtained by Illumina HiSeq sequencing, and the resultant clean data was used for subsequent analysis (Karlsson et al., 2013). To remove host sequences from the samples, reads were filtered by comparison against the host database using Bowtie2 with the following parameter settings: --sensitive, −*I* 200, −*x* 400 (Karlsson et al., 2012; Scher et al., 2013).

### 2.5. Metagenome assembly

After the above preprocessing steps, metagenome assembly was performed using the MEGAHIT software. The assembly parameters were as follows: --*k*-min 35, --*k*-max 95, --*k*-step 20, --min-contig-len 500. For each sample, the preprocessed clean data was compared against the assembled contigs to identify paired-end reads that were not utilized, using Bowtie2 with the following parameters: −*I* 200, −*x* 400 (Karlsson et al., 2013; Nielsen et al., 2014). Unutilized reads from each sample were combined for mixed assembly, with the same assembly parameters as for the single samples.

### 2.6. Gene prediction and abundance analysis

Based on the assembled contigs (≥500 bp) from initial and mixed assembly, MetaGeneMark was used with default parameters to predict ORFs (open reading frames), and after initial analysis, predicted genes shorter than 100 nt were filtered out (Mende et al., 2012; Li et al., 2014; Oh et al., 2014; Qin et al., 2014). The filtered ORF prediction results of all samples (including mixed assembly) were then combined, and CD-HIT (Li and Godzik, 2006; Fu et al., 2012) was used to remove redundancy and obtain a nonredundant initial gene catalog (with each gene defined by the longest nonredundant continuous nucleic acid sequence corresponding to that gene). The following parameter settings were used: −*c* 0.95, −*g* 0, −*AS* 0.9, −*g* 1, −*d* 0 (Li et al., 2014; Qin et al., 2014). By default, 95% identity and 90% coverage were used for clustering; and as mentioned above, the longest sequence for each gene was selected as the representative sequence. Next, Bowtie2 was used to compare the clean data for each sample against the initial gene catalogue (Li et al., 2014), and the number of reads per gene was calculated for each sample, using the following settings: --end-to-end, --sensitive, −*I* 200, −*x* 400. Genes with ≤2 reads in each sample were filtered out to obtain a final gene catalog (of Unigenes) that was used for subsequent analysis. Based on the number of reads and gene length, an abundance value was calculated for each gene in each sample, according to the following equation (Cotillard et al., 2013; Le Chatelier et al., 2013; Villar et al., 2015), where R is the number of reads compared to the gene, and *L* is the length of the gene.

### 2.7. Species annotation

To generate species annotations, the DIAMOND software (Buchfink et al., 2015) was employed to compare Unigene sequences against those of bacteria, fungi, archaea, and viruses from the NCBI NR database, using the lowest common ancestor (LCA) algorithm with the BLASTP search protocol and *E*-value ≤10^−5^. Note that because each sequence may give multiple comparison results, many different species classifications can be obtained. To ensure biological significance, the MEGAN software (Huson et al., 2011) was used again with the LCA algorithm to obtain final species annotation information for the sequences. Based on the results of LCA-based annotation and the gene abundance values, species abundance information was derived for each sample at each taxonomic level (i.e., phylum and genus), and a gene number table for each sample at each level (phylum compendium genus) was also obtained. The abundance of a species in an individual sample was taken as the sum of the annotated gene abundance of that species. The gene number of a species in a sample was defined as the number of genes annotated from that species, for all genes with nonzero abundance. In addition, the following procedures were performed based on the abundance tables at each classification level (genus/species of phylum): Krona analysis (Ondov et al., 2011); relative abundance overview; abundance clustering heat map analysis; principal component analysis (PCA); nonmetric multidimensional scaling (NMDS) dimensionality reduction; analysis of similarities (ANOSIM) inter-/intra-group difference analysis (Rao, 1961); and linear discriminant analysis effect size (LEfSe) multivariate statistical analysis of intergroup differential species.

### 2.8. Common functions database annotation

#### 2.8.1. Sequence alignment

Unigenes were compared against functional databases using DIAMOND (with BLASTP, *E*-value ≤10^−5^). To filter the results for each sequence, the DIAMOND parameter “--max-target-seqs 1” was used to retain unique comparison results. Furthermore, the KEGG database is subcategorized into six functional levels (Kanehisa et al., 2006, 2017), while the eggnog (Huerta-Cepas et al., 2016) and CAZy databases (Cantarel et al., 2009) have three such levels. The comparison results therefore allowed the relative abundance of different functional levels to be calculated (i.e., where the relative abundance of each functional level is equal to the sum of the relative abundance of genes annotated at that functional level). Based on the results of functional annotation and the gene abundance table, the gene number table of each sample at each classification level was obtained. The number of genes with a certain function in a sample was taken as the number of genes with a nonzero abundance among the genes annotated with that function. Based on the gene abundance tables at each classification level and the number of annotated genes, the following procedures were carried out: relative abundance overview; abundance clustering heat map analysis; PCA; NMDS dimensionality reduction; ANOSIM intergroup difference analysis based on functional abundance; and metabolic pathway comparative analysis. Also, Metastate and LEfSe analyses of functional differences between groups were performed.

#### 2.8.2. Resistance gene annotation

The CARD database^1^ was used in conjunction with the accompanying Resistance Gene Identifier (RGI) software to search for antibiotic resistance ontology (ARO) associations among the Unigenes (using RGI’s built-in BLASTP function, with the default *E*-value of <10^−30^) (McArthur et al., 2013). The relative abundance of each ARO was calculated based on RGI and the Unigene abundance information. This allowed construction of an abundance histogram, abundance clustering heat map, and abundance distribution circle map to visualize ARO differences between groups, in terms of resistance genes (i.e., Unigenes annotated with ARO) and species attribution analysis of resistance mechanisms.

### 2.9. Mass spectrometry conditions

All samples were acquired by the LC-MS system followed machine orders. The analytical conditions were as follows, UPLC: column, Waters ACQUITY UPLC HSS T3 C18 (1.8 μm, 2.1 mm × 100 mm); column temperature, 40°C; flow rate, 0.4 mL/min; injection volume, 2 μL; solvent system, water (0.1% formic acid): acetonitrile (0.1% formic acid); The column was eluted with 5% mobile phase B (0.1% formic acid in acetonitrile) at 0 min followed by a linear gradient to 90% mobile phase B (0.1% formic acid in acetonitrile) over 11 min, held for 1 min, and then come back to 5% mobile phase B within 0.1 min, held for 1.9 min.

### 2.10. Statistical analysis

The original data file acquisited by LC-MS was converted into mzML format by ProteoWizard software. Unsupervised PCA (principal component analysis) was performed by statistics function prcomp within R.^2^ The HCA (hierarchical cluster analysis) results of samples and metabolites were presented as heat maps with dendrograms. For two-group analysis, differential metabolites were determined by VIP (VIP >1) and *p*-value (*p*-value <0.05, Student’s t test). Identified metabolites and annotated metabolites were annotated and mapped using KEGG Compound and Pathway database (http://www.kegg.jp/kegg/compound/ and http://www.kegg.jp/kegg/pathway.html).

The data was processed using SPSS 22.0 and values are expressed as (*x ® * ± *s*). The t-test was used for pairwise comparisons between groups if the data met both normality and chi-square; if not, the rank sum test was used. One-way ANOVA was used to compare multiple groups. Differences where *p* < 0.05 were regarded as statistically significant.

## 3. Results

### 3.1. Gene prediction and operational taxon abundance analysis

**Figure 1:**
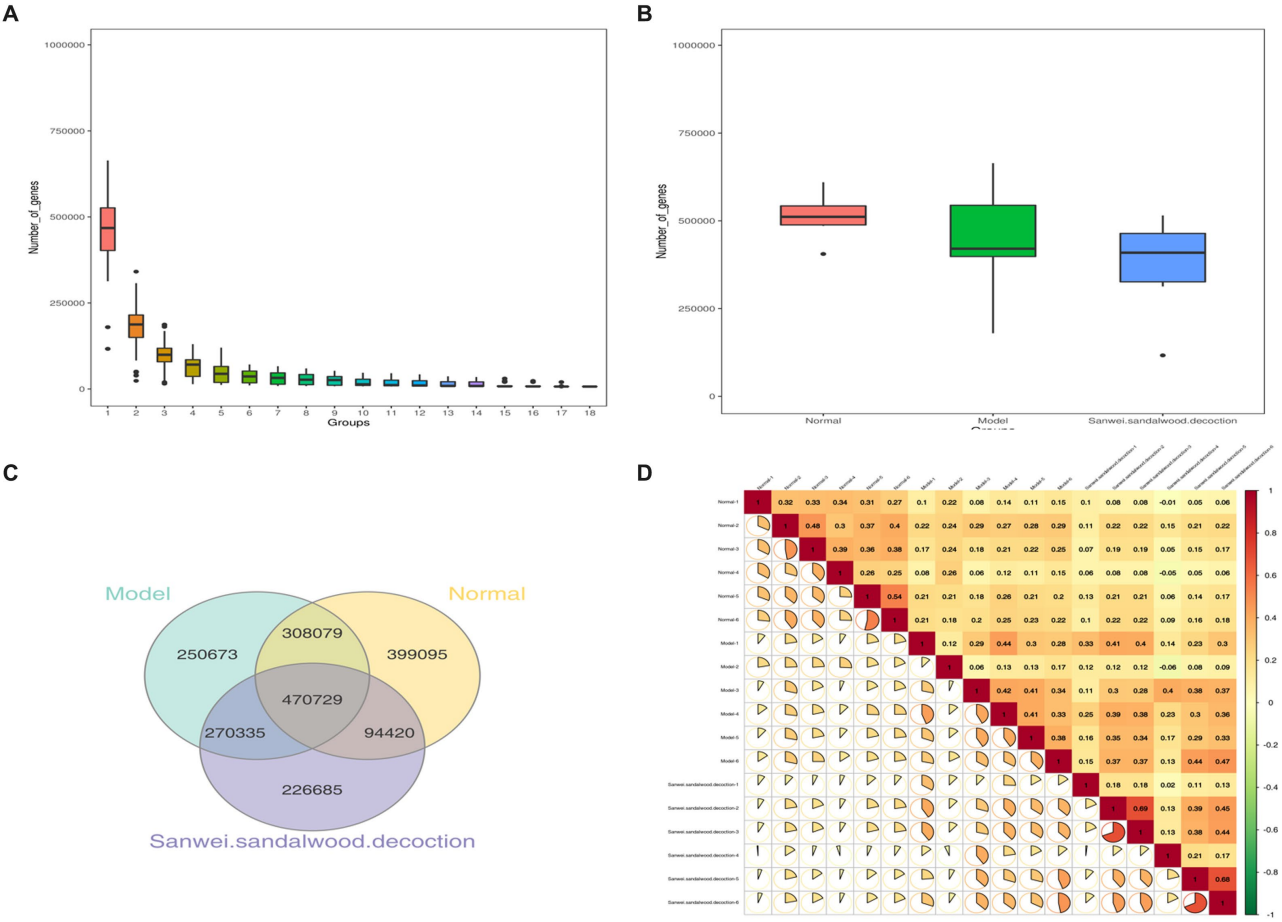
**(A,B)**: Dilution profile of core-pan gene; box plot of gene number differences between groups; **(C)**: Clustering of intestinal flora OTUs in each group of rats Venn diagram; **(D)**: Heat map of correlation coefficients between samples.

Basic statistics on the gene catalog are presented in Table 1. Using the gene abundance information, core genome and pan-genome analyses were performed, along with intersample correlation analysis and Venn diagram construction. As depicted in Figure 1A, the core and pan-genome analyses revealed the number of genes after sample dilution. The sparse curve tends to flatten out and approach with increasing data, indicating that the collected samples were suitable for subsequent bioinformatics analysis. The number of genes differed between groups, and there was also variability between groups (Figure 1B). The Venn diagram (Figure 1C) shows that there were 778,808 common genes between the blank and model groups, while 49,315 genes were lost and 521,008 were gained in the model group relative to the blank group, suggesting that heart failure induces changes in the number of microbial genes found in feces. Compared with the model group, this value was also significantly affected by treatment with Sanwei sandalwood decoction. Herein, in the rat heart failure model, the number of genes specific to the intestinal flora increased after the therapeutic intervention. Moreover, we determined the correlation between samples to assess the degree of similarity between them, i.e., in terms of gene expression levels. As shown in Figure 1D, the correlation coefficients for all pairwise comparisons were <1, indicating a low degree of similarity and implying a high number of differential genes between experimental groups.

**Table 1:** Unigenes basic information statistics.

List	Unigenes
ORFs No.	3,199,158
Integrity:all	1,500,838 (46.91%)
Integrity:none	209,760 (6.56%)
Integrity:end	847,236 (26.48%)
Integrity:start	641,324 (20.05%)
Total Len.(Mbp)	1874.82
Average Len.(bp)	586.04
GC percent	49.08

### 3.2. Relative abundance and annotation of rat fecal microorganisms

#### 3.2.1. Results of species relative abundance analysis

**Figure 2:**
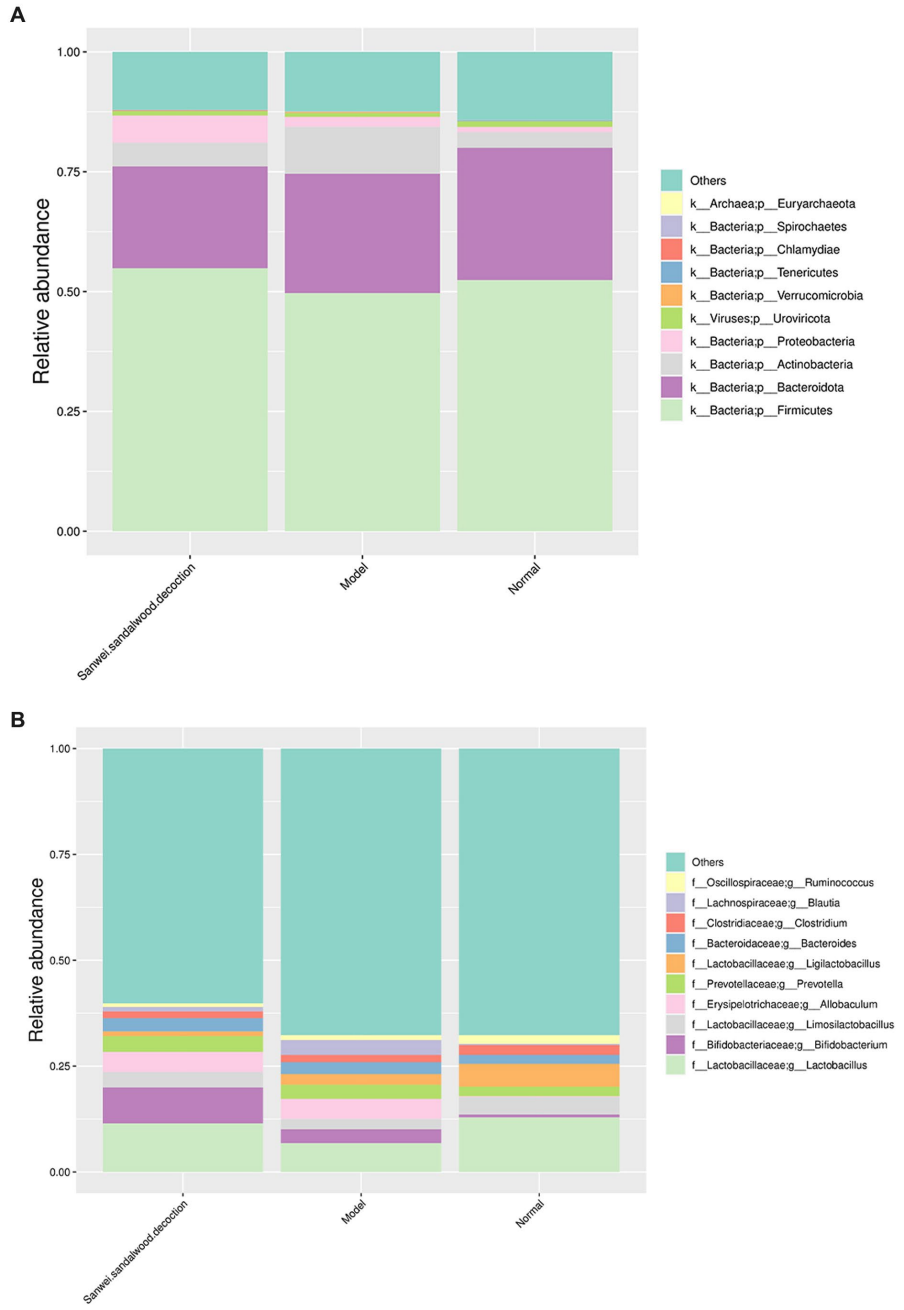
Histogram of relative abundance of species at the phylum level and genus level (group). **(A)** Histogram of relative abundance at the phylum level; **(B)** Histogram of relative abundance at the genus level. The horizontal axis indicates the name of the sample; the vertical axis indicates the relative proportion of species annotated to a type; the species categories corresponding to each colour block are shown in the legend on the right.

To visualize the composition and relative abundance of species at different taxonomic levels of phylum and genus, the annotated sequence information represented by the clustered operational taxon abundance (OTUs) was used to plot cumulative bar charts for the top 10 species in relative abundance, for all samples. At the phylum level (Figure 2A), all groups of rat fecal samples were dominated by Firmicutes, Bacteroidota, Actinobacteria, and Proteobacteria, but there were some differences in proportion and species composition between groups. In the model group, the relative abundance of Firmicutes decreased and that of Actinobacteria and Proteobacteria increased, compared with the blank group. In the treatment group, the relative abundance of Firmicutes and Proteobacteria increased, while that of Bacteroidota and Actinobacteria decreased, compared with the model group. As indicated in Figure 2B, the abundance of samples in each group changed as the taxonomic hierarchy of species changed, and the differences between groups were more pronounced.

At the genus level, the microbial community composition in all experimental groups of rat fecal samples was dominated by *Lactobacillus*, *Bifidobacterium*, *Limosilactobacillus*, *Allobaculum*, *Prevotella*, and *Ligilactobacillus* spp., but similarly to the phylum level, some differences in relative abundance were apparent between groups. In terms of species composition, the relative abundance of *Lactobacillus*, *Limosilactobacillus*, and *Ligilactobacillus* spp. decreased, while that of *Bifidobacterium*, *Allobaculum*, and *Prevotella* spp. increased, in the modelgroup compared with the blank group. For the treatment group, the relative abundance of *Lactobacillus*, *Bifidobacterium*, and *Limosilactobacillus* spp. increased compared with the modelgroup, while that of *Ligilactobacillus* spp. decreased.

#### 3.2.2. Cluster analysis of the relative abundance of the number of annotated genes, and dimensionality reduction of species abundance

**Figure 3:**
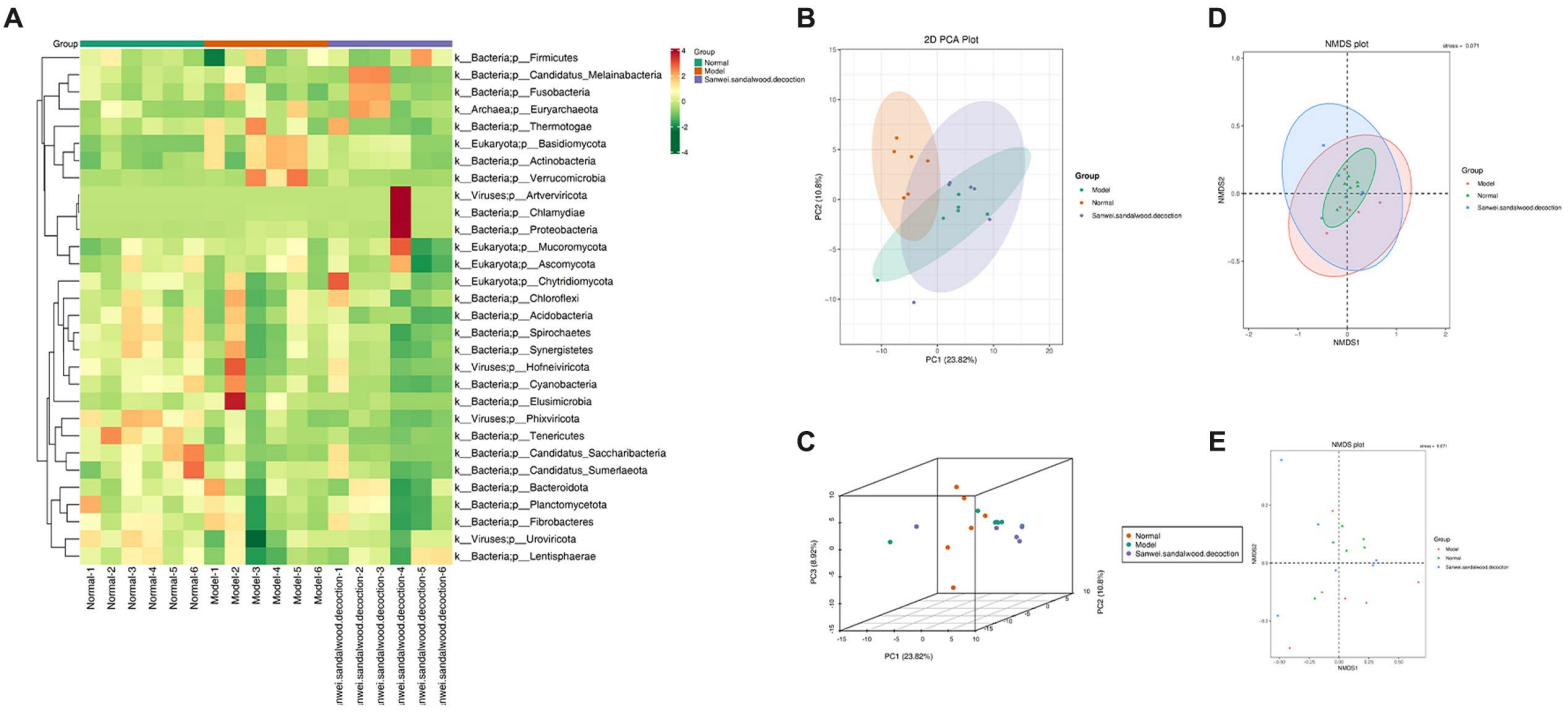
**(A)**: Heat map of gate level abundance clustering; **(B-E)**: Presentation of NMDS results for species based on gate level, presentation of PCA results for species based on gate level.

The top 35 genera (in terms of abundance and information on their abundance in each sample) from the relative abundance tables, at different taxonomic levels, were used for heat mapping and clustering at the species level. This facilitated visualization of the results and aided information discovery, thereby enabling identification of species that were more aggregated in the samples (Figure 3A). To aid the interpretation of results, PCA, NMDS plot were used as a statistical approach, since it is able to reduce multiple variables into a few principal components through dimensionality reduction. It is one of the most important methods for achieving this, and when applied to the analysis of gut microorganisms, PCA therefore focuses on finding the most relevant combination of species to represent the overall gut microbiome. In the blank group of the current experiment, it can be seen that the samples were clustered and well differentiated. Additionally, in both the model and Sanwei sandalwood decoction groups, the samples were generally discrete and differentiated, but one abnormal sample was seen in each group (Figures 3B–E). This may reflect individual variation in intestinal microorganisms, or perhaps it was a consequence of the small sample size.

### 3.3. Analysis of species differences across groups

#### 3.3.1. Clustering analysis of samples based on species abundance

**Figure 4:**
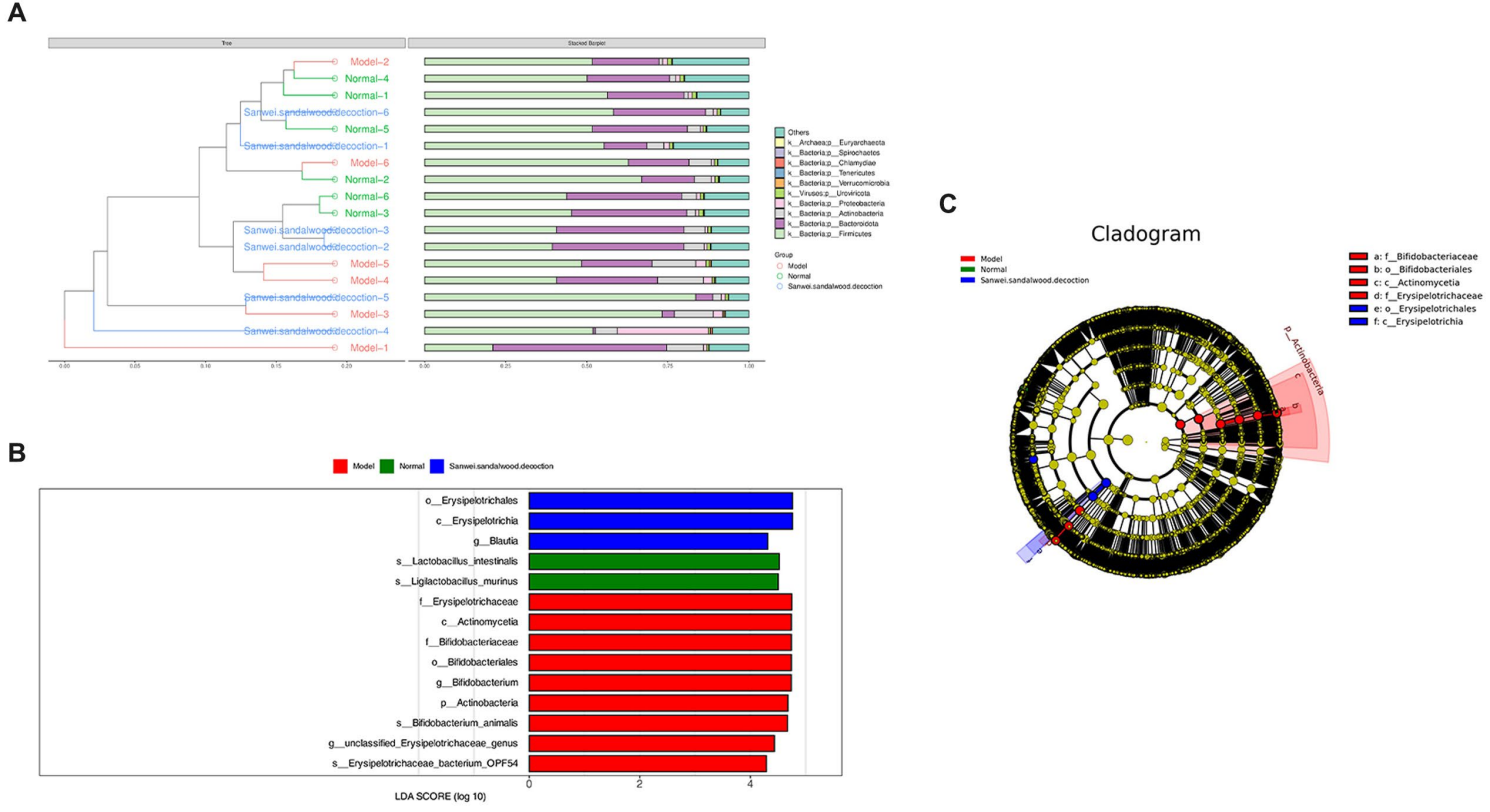
**(A)** In the BrayCurtis distance-based clustering tree diagram, the Bray-Curtis distance clustering tree structure is shown on the left side; on the right side, the relative abundance distribution of species at the gate level for each sample is plotted. **(B)** Histogram of the distribution of LDA values for differing species. **(C)** Evolutionary branching diagrams for divergent species, circles radiating from inside to outside represent taxonomic levels from phylum to genus (or species). Species with no significant differences are uniformly coloured yellow, and differential species Biomarker follows the group for colouring, with red nodes indicating microbial taxa that play an important role in the red group and green nodes indicating microbial taxa that play an important role in the green group.

The Bray–Curtis distance is often used to characterize the degree of similarity between samples, and is one of the most commonly used distance metrics in systematic clustering. Based on the tables of gene abundance for the experimental samples, a Bray–Curtis distance matrix was used to perform intersample clustering. The results are presented with integration of the relative abundance of species at the gate level, for each group (Figure 4A).

#### 3.3.2. LEfSe analysis of variance

The LEfSe analysis of variance method uses the Kruskal–Wallis rank sum test and the Wilcoxon rank sum test to screen for species with significant differences in abundance between groups. It then uses linear discriminant analysis to downscale the data and assess the influence of species with significant differences, to find those species or biomarkers that most influence the differences between groups. Using the LEfSe online analysis tool, three such species were identified (Figures 4B,C). It is evident that the class Erysipelotrichia, the order Erysipelotrichales, and the genus *Blautia* were enriched in the Sanwei sandalwood decoction group. Furthermore, the class Actinomycetia, the order Bifidobacteriales, the families Erysipelotrichaceae and Bifidobacteriales, the genera *Bifidobacterium* and *Ligilactobacillus*, and the species *Lactobacillus intestinalis* were enriched in the model group.

### 3.4. Analysis of relative abundance and differences in the biological functions of rat fecal microorganisms

#### 3.4.1. Analysis of the relative abundance of functional annotations by class

**Figure 5:**
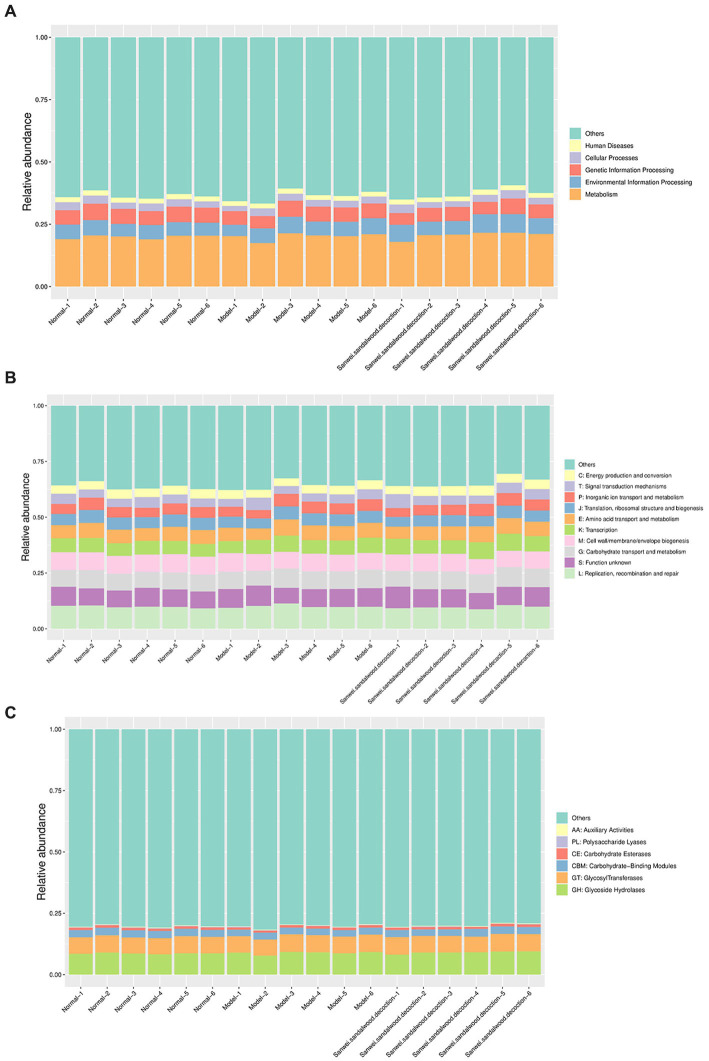
Histogram of relative abundance of functional annotations on level 1.The results of KEGG, eggNOG, and CAZy are shown in order. **(A)**: Histogram of relative abundance of functional annotations in KEGG; **(B)**: Histogram of relative abundance of functional annotations in eggNOG; **(C)**: Histogram of relative abundance of functional annotations in CAZy. The vertical axis indicates the relative proportion of annotations to a functional class; the horizontal axis indicates the sample name; the functional class corresponding to each colour block is shown in the legend on the right.

Analysis using the KEGG PATHWAY database revealed that the KEGG class I metabolic pathways with the highest relative abundance in the rat fecal samples included metabolism, genetic information processing, and environmental information processing (Figure 5A). Through comparative analysis of rat fecal microbiome macrogenomes, based on the eggNOG database, the functional classes replication, recombination, and repair; cell wall/membrane/envelope biogenesis; carbohydrate transport and metabolism; and amino acid transport and metabolism were found to be more abundant in the rat fecal microbiome (Figure 5B). Moreover, comparative macrogenomic analysis using the CAZy database showed that the functional classes of glycoside hydrolases (GHs), glycosyltransferases (GTs), and carbohydrate-binding modules (CBMs) were relatively abundant in the rat fecal microbiome, among the six CAZy functional modules (Figure 5C).

#### 3.4.2. Analysis of the differences in the relative abundance of functions

**Figure 6:**
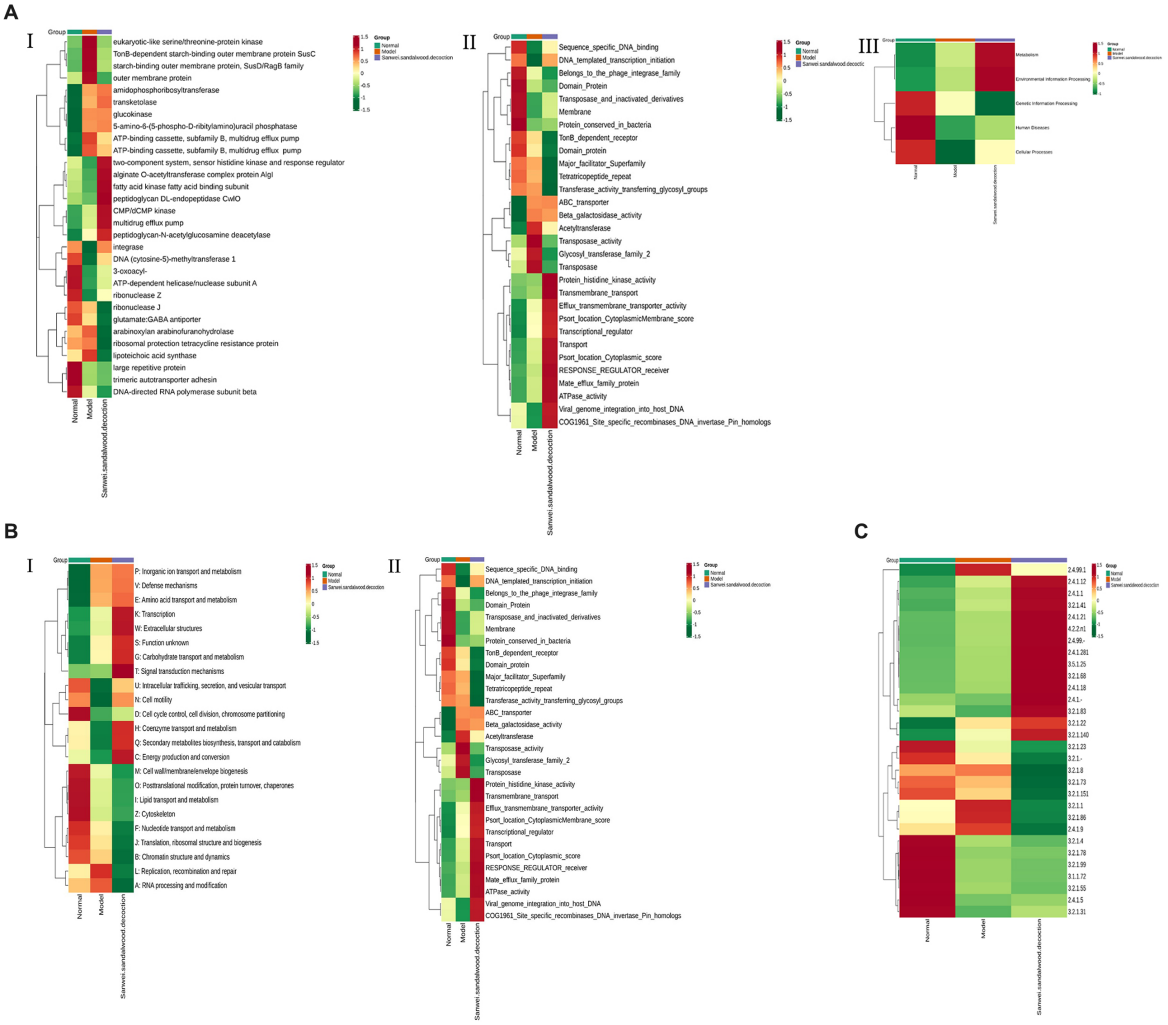
Functional abundance clustering heat map. The results of KEGG, eggNOG, and CAZy are shown sequentially. **(A)**: Functional abundance clustering heat map in KEGG (I is for level 1, II is for level 2, III is for level 3); **(B)**: Functional abundance clustering heat map in eggnog (I is for level 1, II is for level 2); **(C)**: Functional abundance clustering heat map in CAZy. Sample information is shown horizontally; functional annotation information is shown vertically; the clustering tree on the left side of the graph is the functional clustering tree; the values corresponding to the heat map in the middle are the Z-values obtained from normalising the relative abundance of the functions in each row.

The relative abundance of each functional class—such as KEGG, eggNOG, and CAZy protein families—was compared between rat fecal microbiomes, based on the relative abundance distribution of the underlying functional classes that were assigned using each database, and the differences were evaluated for statistical significance. First, cluster analysis was performed on the top 30 KEGG ontology (KO) terms displaying significant differences in relative abundance in the rat fecal microbiome among experimental groups (Figure 6AI). The level 2 categories amino acid metabolism; energy metabolism; environment information processing: signal transduction; carbohydrate metabolism; and lipid metabolism were significantly higher in the Sanwei sandalwood decoction group than in the blank and modelgroups (Figure 6AII). At level 1, it emerged that the cluster analysis was higher in the same group in terms of metabolism and environment information processing, compared with the blank and model groups (Figure 6AIII).

Based on the relative abundance distribution of each functional class that was annotated from the eggNOG and CAZy databases, we evaluated the relationships between rat fecal microbiomes in the different experimental groups, to assess which functional classes were enriched in each group. In this macrogenomic analysis, assessment of eggNOG functional annotations indicated variations in level 1 classification; level 2 classification; secondary metabolite biosynthesis; transport and catabolism; energy production and conversion; and transmigration. Moreover, energy production and conversion; transcription; amino acid transport and metabolism; signal transduction mechanisms; and nucleotide transport and metabolism were significantly higher in the feces of rats in the Sanwei sandalwood decoction group than for those in the blank and modelgroups. The relative abundance of cell wall/membrane/envelope biogenesis; coenzyme transport and metabolism; posttranslational modification (Figures 6B,C). Functional annotation analysis using the CAZy database showed that CBMs, GTs, polysaccharide lyases (PLs), GHs, and carbohydrate esterases (CEs) had significantly higher relative abundance in the fecal microbiota of rats in the treatment group (Sanwei sandalwood decoction) groups, compared with the blank and model groups.

### 3.5. Resistance genes are prevalent in both human gut microbes and other environmental microbes

Misuse of antibiotics has induced irreversible changes in human and environmental microbial communities, posing new risks to human health and ecological systems. The study of antibiotic resistance genes (ARGs) has therefore received considerable attention from researchers (Guo et al., 2018). In recent years, the CARD resistance gene database has been developed to assist these efforts. It has the advantages of comprehensive information coverage, user-friendliness, and timely updates and maintenance (Jia et al., 2017). Here, it was used to analyze the abundance of AROs and produce a bar chart of abundance, a heat map of abundance clusters, and a map of abundance circles, as well as identifying ARO differences between groups. It also provided information on the species affiliation of resistance genes (i.e., Unigenes annotated to AROs). The details are shown in Supplementary Figure S1.

To visualize the overall distribution of ARO abundance in each group, the top 10 most abundant AROs were selected and plotted in an overview circle (Supplementary Figures S2, S3A). Additionally, the top 30 AROs were used to generate an abundance clustering heat map; and to test whether between-group differences were significantly greater than within-group differences, ANOSIM analysis of the abundance of resistance genes was performed (Supplementary Figure S3B). To further examine differences between sample groups, box plots were made of differences in the number of resistance genes and the number of AROs between groups (Supplementary Figure S3C).

### 3.6. Nontargeted metabolomics

#### 3.6.1. Principal component analysis of subgroups

**Figure 7:**
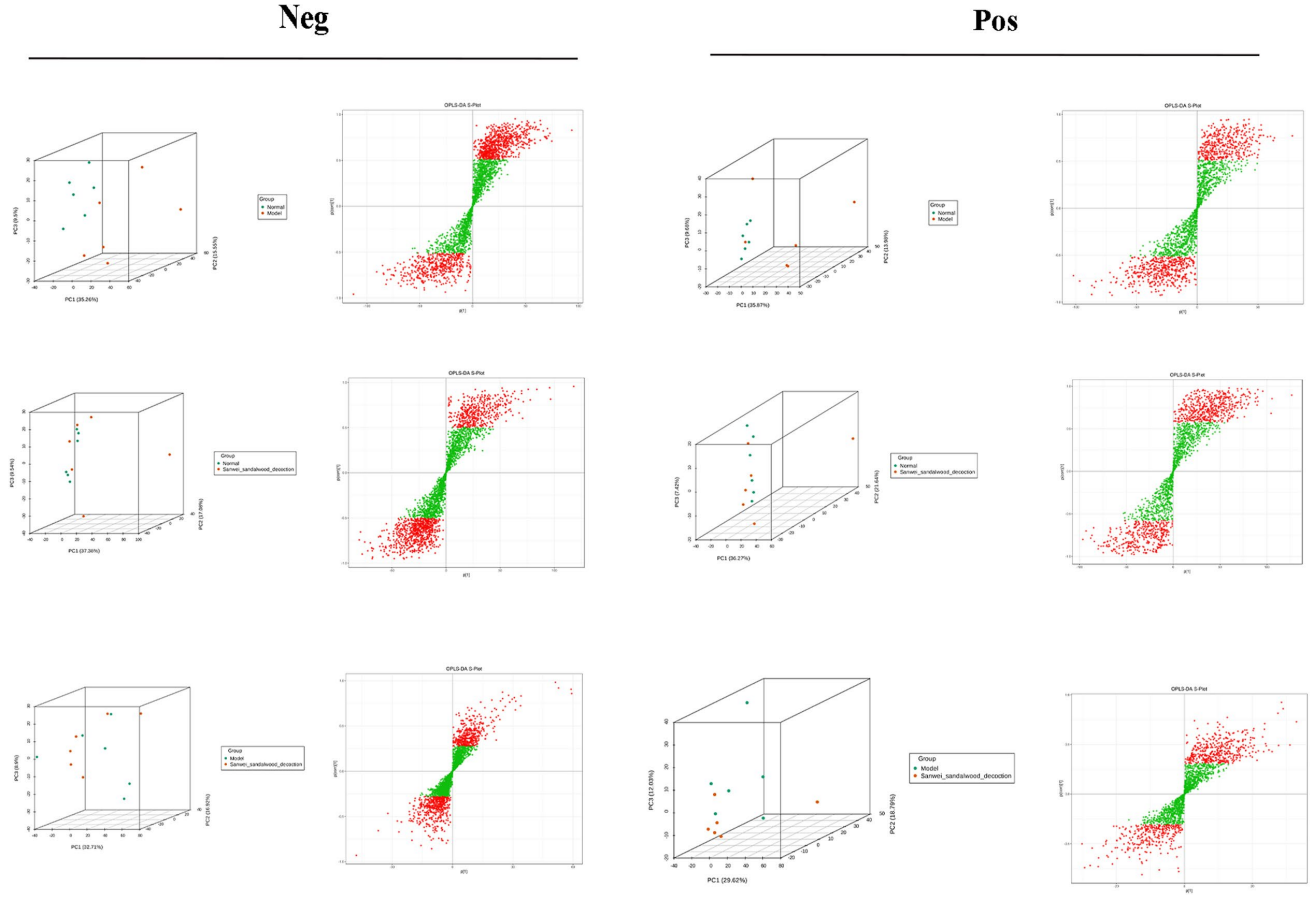
PCA vs. OPLS-DA plots between groups in the negative and positive.

The samples of the subgroups for variance comparison were first subjected to principal component analysis and orthogonal partial least squares discriminant analysis in order to observe the magnitude of variability between the variance subgroups and between the samples within the groups, which is conducive to the search for variance metabolites. From the Figure 7, the group can be clearly distinguished, indicating that there is a difference between the groups, and it can be seen that the position of the Sanwei sandalwood decoction group is close to the normal group, indicating that it has a good regulating effect on heart failure.

#### 3.6.2. Differential metabolite screening

**Figure 8:**
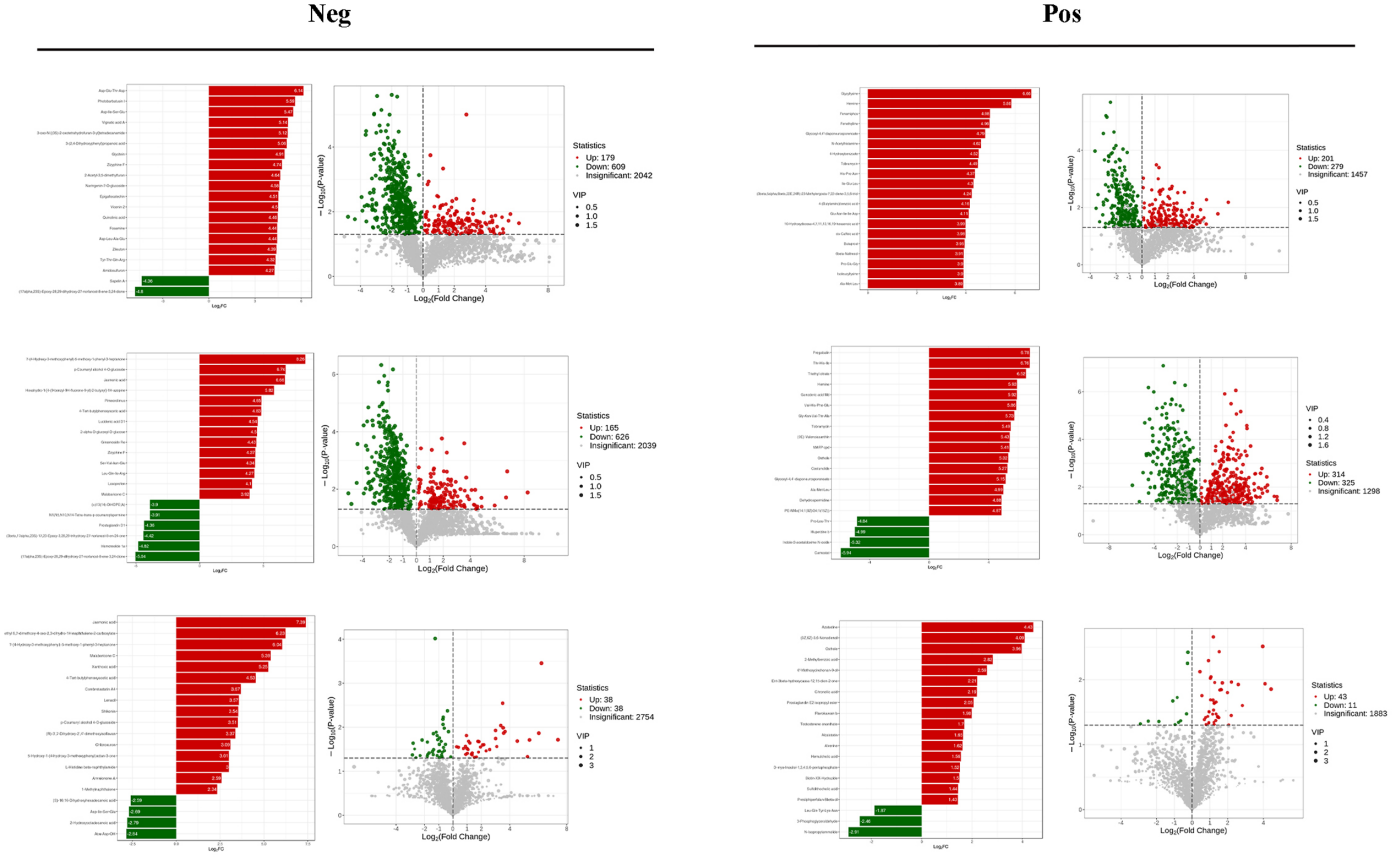
Differential multiplicity histograms and volcano plots for the first 20 metabolite results for each subgroup comparison.

**Figure 9:**
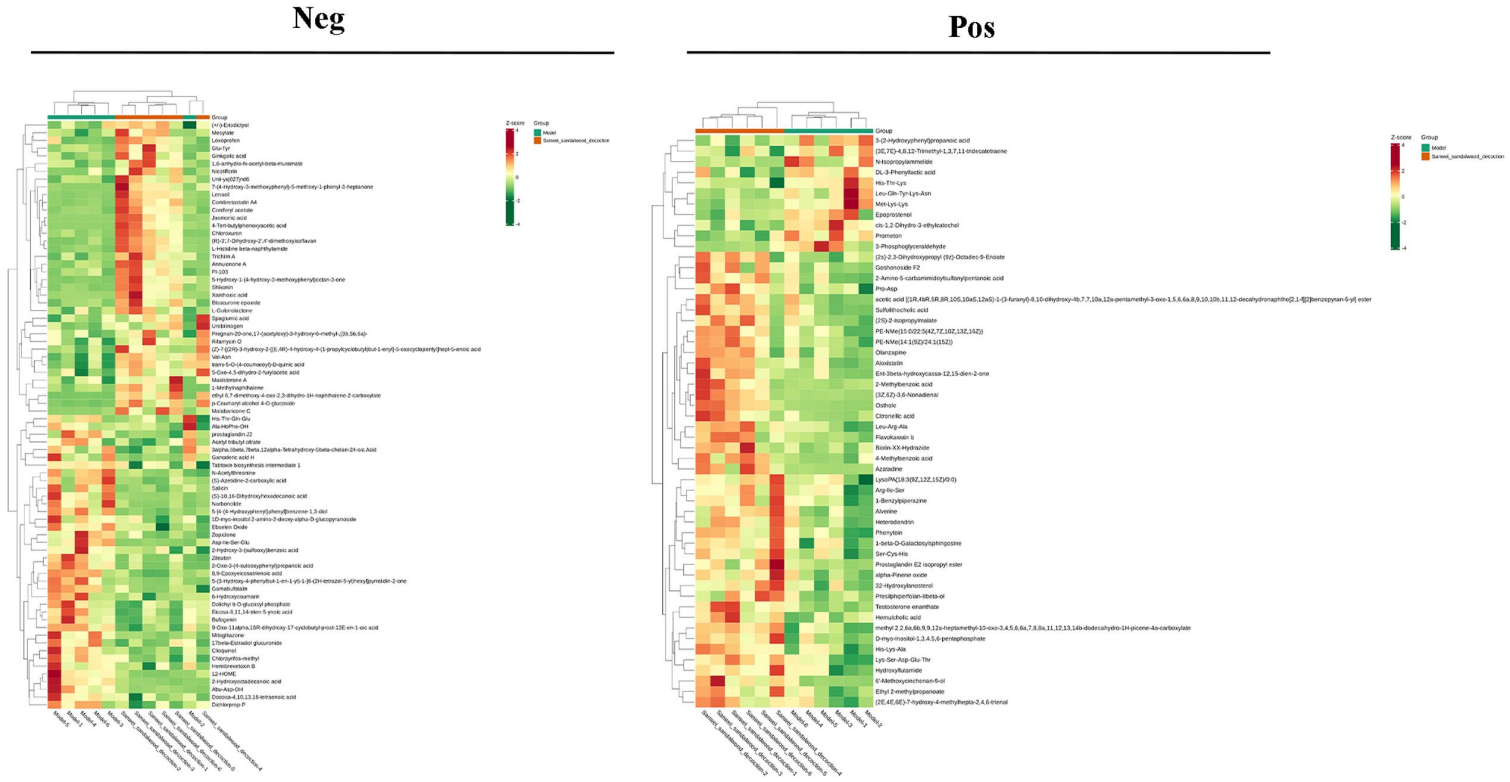
Heat map of differential metabolites between the model group and the Sanwei sandalwood decoction group in the negative and positive.

VIP >1 metabolites were chosen. It is commonly accepted that metabolites with VIP >1 are substantially different. VIP value shows the degree of the influence of between-group differences of the corresponding metabolite in the categorical discrimination of each group of samples in the model. The student’s *t*-test *p*-value 0.05-selected metabolites. When there was a statistically significant difference between groups, metabolites were regarded as differing considerably. Table 2 displays some of the results of the differential metabolite screening. Sample-specific subgroups were pooled to assess the multiplicity-of-difference changes in quantitative metabolite information across subgroups following qualitative and quantitative analysis of the discovered metabolites. Along with multiplicity of difference bar charts and volcano plots, the top 20 metabolite findings for each subgroup comparison are displayed (Figure 8). The original relative content of differential metabolites obtained by using the screening standard identification was standardised by rows using unit variance scaling (UV), and the heat map was plotted by R software. The results are shown in Figure 9 and Supplementary Figure S4. These differential metabolites may eventually be involved in potential biomarkers of Sanwei sandalwood decoction cardio-modulation through the gut, laying the groundwork for future research.

**Table 2:** Results of differential metabolite screening.

Index	Compounds	Type
MW0149468	Ganoderic acid H	Down
MW0001649	1-Methylnaphthalene	Up
MW0005526	5-[4-(4-Hydroxyphenyl)phenyl]benzene-1,3-diol	Down
MW0136801	7-(4-Hydroxy-3-methoxyphenyl)-5-methoxy-1-phenyl-3-heptanone	Up
MW0144012	8,9-Epoxyeicosatrienoic acid	Down
MW0138813	Loxoprofen	Up
MEDP1238	Conifery-l-acetate	Up
MW0143316	5-Hydroxy-1-(4-hydroxy-3-methoxyphenyl)octan-3-one	Up
MW0158037	Trichilin A	Up
MW0105417	Acetyl tributyl citrate	Down
MW0141802	1D-myo-inositol 2-amino-2-deoxy-alpha-D-glucopyranoside	Down
MW0108209	Mesylate	Up
MW0152664	L-Histidine beta-naphthylamide	Up
MW0011982	(S)-10,16-Dihydroxyhexadecanoic acid	Down
MW0159254	Val-Asn	Up

#### 3.6.3. Metabolite function annotation

**Figure 10:**
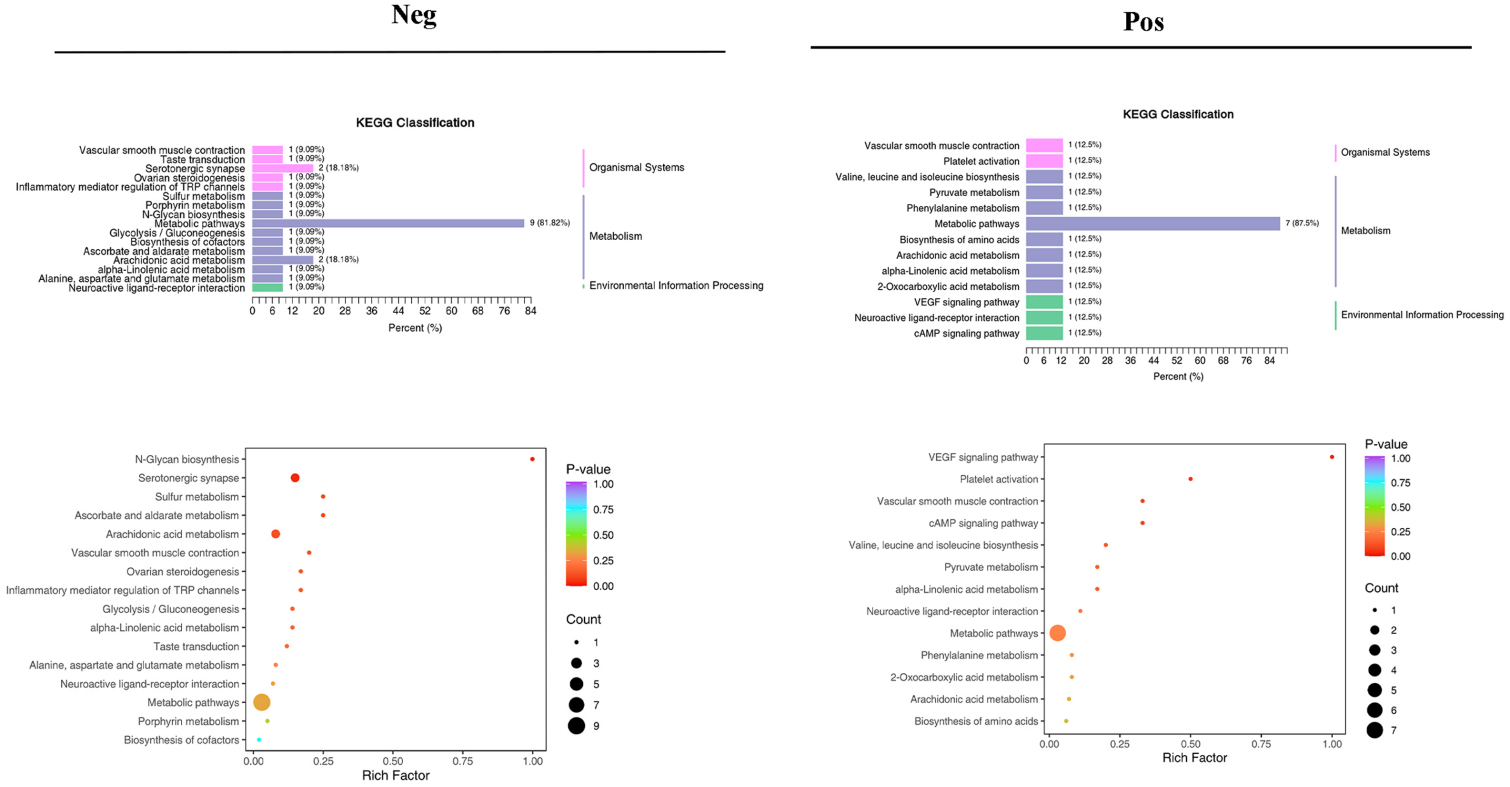
Results of KEGG pathway enrichment analysis of differential metabolites in the negative and positive.

Different pathways are formed inside the body as a result of interactions between metabolites. The KEGG database was used to annotate various metabolites, and the findings were categorized in accordance with the KEGG pathway types, as shown in the accompanying image. KEGG pathway enrichment was performed based on differential metabolite results, and the size of the dots in the graph represents the number of differential significant metabolites enriched to the corresponding pathway. The top 20 pathways ranked by *p*-value were selected and shown from smallest to largest (Figure 10; Supplementary Figure S5).

## 4. Discussion

The human body contains about 10–100 trillion microorganisms, the majority of which are bacteria (Ursell et al., 2012). This is a huge number that far exceeds the number of somatic cells, and research in recent years has increasingly demonstrated the important role of the gut microbiome for the host, in both health and disease. Under physiological conditions, the intestinal flora is in relative homeostasis, but when this balance is disturbed, pathogenic microorganisms can lead to host diseases including inflammatory bowel disease and colorectal cancer, as well as nonintestinal disorders such as obesity, diabetes, autism, and cardiovascular disease (Jia et al., 2019). Given the key regulatory role of gut microorganisms in a variety of disease areas, researchers have increased their interest in—and exploration of—new therapeutic approaches and strategies based on targeting the intestinal flora.

As outlined above, heart failure is recognized as a multifactorial systemic disease with extremely complex pathology, yet one important mechanism that informs and explains the associated pathological changes is the inflammatory response. It has previously been shown that the development of heart failure leads to intestinal mucosal damage and flora displacement, and that this dysregulated microbiome causes numerous adverse effects in late-stage disease by increasing expression of regulatory factors that mediate the inflammatory response (Guo et al., 2018; Yu and Jinmin, 2019). In view of this, we hypothesized that intervention strategies capable of modulating the composition and diversity of the gut flora, enhancing the intestinal barrier, reducing intestinal permeability, intervening in microbial metabolite changes, and reducing the levels of proinflammatory factors would constitute effective treatments for heart failure.

Moreover, in the context of modern microecological research, the gut is considered to be the largest organ of the body. Intestinal microbial homeostasis has a beneficial effect on normal metabolism, while conversely, an imbalance in the gut flora can negatively impact the endocrine, immune, neurological, and even mental functions of the host, provoking a series of pathophysiological changes that can manifest as cardiovascular disease; the so-called “cardio-intestinal” axis adjustment.

### 4.1. Relative abundance of intestinal flora species and abundance clustering analysis

The relative abundance of the genera *Bacteroides* and *Prevotella*, and the phylum Bacteroidetes, was significantly reduced in the intestinal flora in the rat model of heart failure, while the relative abundance of the genera *Bacteroides*, *Clostridium*, and *Lactobacillus*, and the order Actinomycetales, was markedly increased. After treatment with Sanwei sandalwood decoction, the relative abundance of the phylum Bacteroidota, and the genera *Aspergillus* and *Prevotella*, increased significantly in the intestinal flora, while that of *Lactobacillus* spp. decreased. The intervention also led to a significant reduction in the relative abundance of *Aspergillus* spp. and the phylum Bacteroidota, while balancing the relative abundance of *Lactobacillus* spp. and other genera that had been altered by heart failure. The relative abundance of *Phyllobacterium* spp. in the intestinal flora of rats after heart failure, and the least clustered abundance of these species, were significantly rescued after the treatment intervention; and the gut microbiota became more enriched in *Phyllobacterium* spp., *Lactobacillus* spp., and *Bifidobacterium* spp. These are all important probiotic species in the intestinal flora that play key roles in metabolic processes *in vivo*.

### 4.2. Functional annotation distribution and variance analysis

The metabolic functional classes had the highest relative abundance in the intestinal flora, among KEGG class I pathways. Moreover, carbohydrate transport and metabolism, and amino acid transport and metabolism, were more abundant in the gut microbiota of all three experimental groups. Of the six protein functional modules in the CAZy database, the classes GHs, GTs, and CBMs had higher relative abundance; while KO clustering analysis revealed that the Sanwei sandalwood decoction group had a greater degree of energy metabolism, carbohydrate metabolism, amino acid metabolism, and membrane transport compared with the model group. Based on eggNOG functional annotation, the processes of biosynthesis; transport and catabolism of secondary metabolites; energy production and conversion; transcription; amino acid transport and metabolism; signal transduction mechanisms; and nucleotide transport and metabolism were more abundant in the treatment group. The intestinal flora of rats in the dispersion group was significantly higher than that in the model group. In summary, the most abundant functional annotations in the intestinal flora of rats with heart failure, and treated with Sanwei sandalwood decoction, were mainly distributed among CBMs/carbohydrate metabolism, amino acid metabolism, nucleotide transport, energy metabolism, and GTs.

### 4.3. Analysis of untargeted metabolomics in heart failure

Metabolomics enables qualitative and quantitative assessment of metabolites in living organisms (Guo, 2021). Metabolites are downstream of gene transcription and protein translation and are closely related to phenotypes, especially for multifactorial diseases. In recent years, metabolomics has been increasingly used to identify heart failure biomarkers (Laíns et al., 2019). Compared with conventional biomarkers, metabolomics is more accurate in assessing heart failure-related metabolic disorders, and metabolite changes are more conducive to assessing the prognosis of heart failure (Cheng et al., 2015; Lanfear et al., 2017). Plasma-targeted surrogomics studies in heart failure patients with reduced ejection fraction identified 13 metabolites independently associated with survival, further demonstrating the promise of metabolic impairment and metabolomics in assessing heart failure phenotype and risk stratification and identifying therapeutic targets. In this study, PCA and OPLS-DA S-PLOT found that the three groups were clearly distinguished, while the position of the Sandalwood soup group tended to be the normal group, which could improve the metabolism of heart failure, and the classification of KEGG differential metabolites in enrichment analysis showed that Sandalwood soup may regulate heart failure by regulating 15 metabolites such as Loxoprofen, Conifery-l-acetate, and Trichilin A. It can be concluded that arachidonic acid metabolism pathway occupies an important proportion in the pathway classification chart, and it is known that arachidonic acid (AA) is an important long-chain hyperunsaturated fatty acid in organisms, and its metabolism plays an important role in the occurrence and development of myocardial fibrosis (Zhang et al., 2018). Experiments further show that the issue of the possible effect of Sanwei sandalwood decoction on the diversity of intestinal flora in the body by regulating differential metabolites, such as increasing the expression of AA, is also worth further exploration.

### 4.4. Others

It is already established that ARGs change in response to variations in the microbiome (Zhao et al., 2021), and that reductions in ARGs generally correlate with reductions in the bacterial community. In particular, research on β-lactam antibiotic resistance genes (ARGs) is an important area of study that cannot be ignored. In this work, resistance genes against other antibiotic classes—such as tetracyclines, macrolides, and quinolones—were identified less frequently. However, in a finding that has not been previously reported, ARGs for some commonly used disinfectants and antiseptics (*triC* and *qacH*) were detected in this study.

## 5. Conclusion

In conclusion, the intestinal flora plays a key regulatory role in the development of heart failure, and its regulation has therefore become a promising therapeutic strategy for the disease. In this study, we used macrogenomic sequencing of fecal samples to investigate the effects of Sanwei sandalwood decoction on the composition, diversity, and structure of the gut microbiota in a rat model of heart failure. The flora composition and structure were altered in the intervention group to some extent, but our analysis also revealed species with differential and predicted functional advantages. These are of interest in designing improved interventions, and consequently represent a valuable reference point with potential for future application.

## Data availability statement

The datasets presented in this study can be found in online repositories. The names of the repository/repositories and accession number(s) can be found in the article/Supplementary material.

## Ethics statement

The experimental protocol and animal use were approved by the Animal Use and Management Ethics Committee at the Animal Experimentation Management Center of Inner Mongolia University of Nationalities (Lot No. NM-LL-2019-10-12-01). The study was conducted in accordance with the local legislation and institutional requirements.

## Author contributions

KM and QW conceived and designed the experiments. KM, TB, PH, Dalintai, and MZ performed the experiments. QW, Surilige, ZX, and QZ analyzed and interpreted the data. TB, QW, and PH contributed reagents, materials, analysis tools or data. KM and TB wrote the paper. QW provided funding acquisition. All authors contributed to the article and approved the submitted version.

## Footnotes

^1^ https://card.mcmaster.ca/

^2^ www.r-project.org
