# Supplementary figures and images for: Sanwei sandalwood decoction improves function of the gut microbiota in heart failure
Source: Front Microbiol. 2023 Oct 19;14:1236749. doi: 10.3389/fmicb.2023.1236749 (PMC10620746; doi:10.3389/fmicb.2023.1236749)

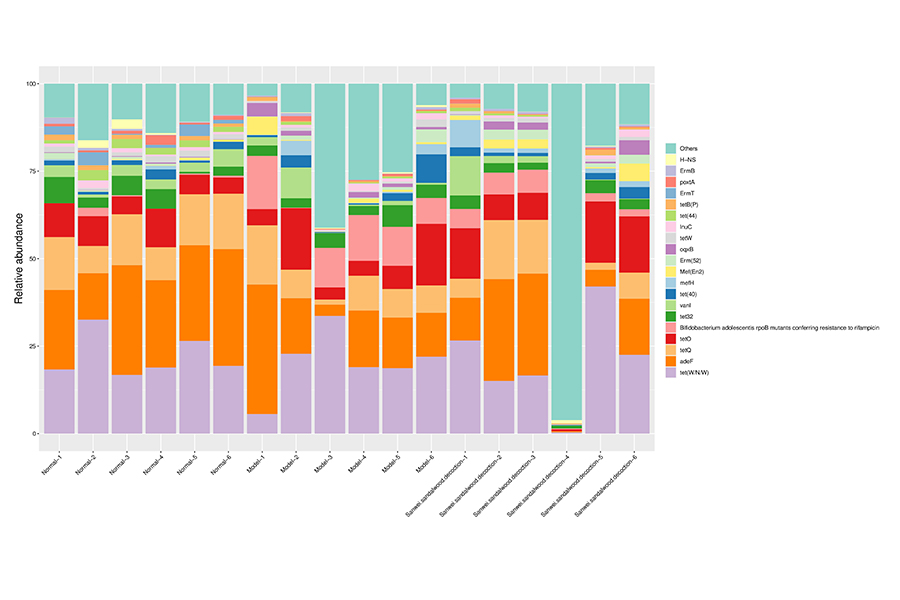

Supplement: Supplementary file 16 [file Image_1.JPEG]

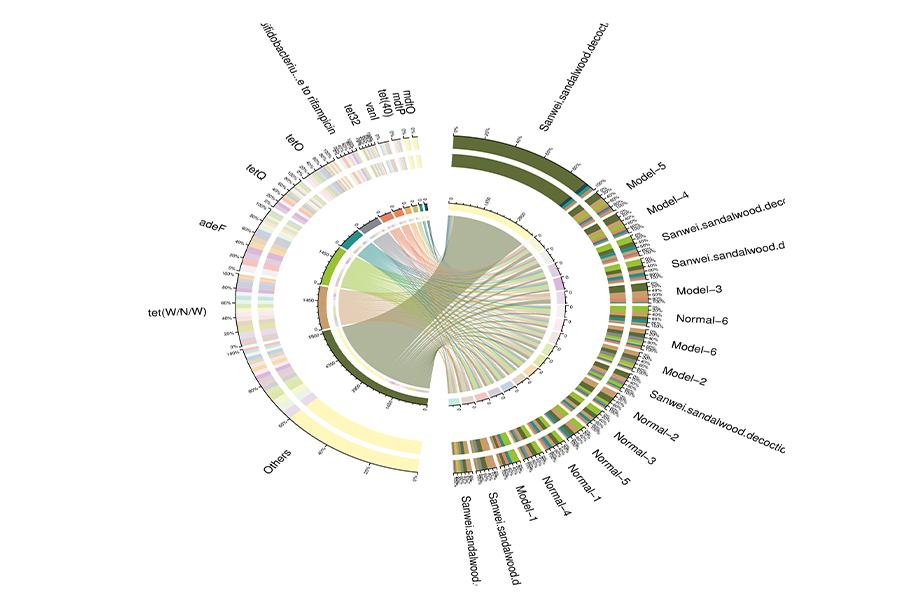

Supplement: Supplementary file 17 [file Image_2.JPEG]

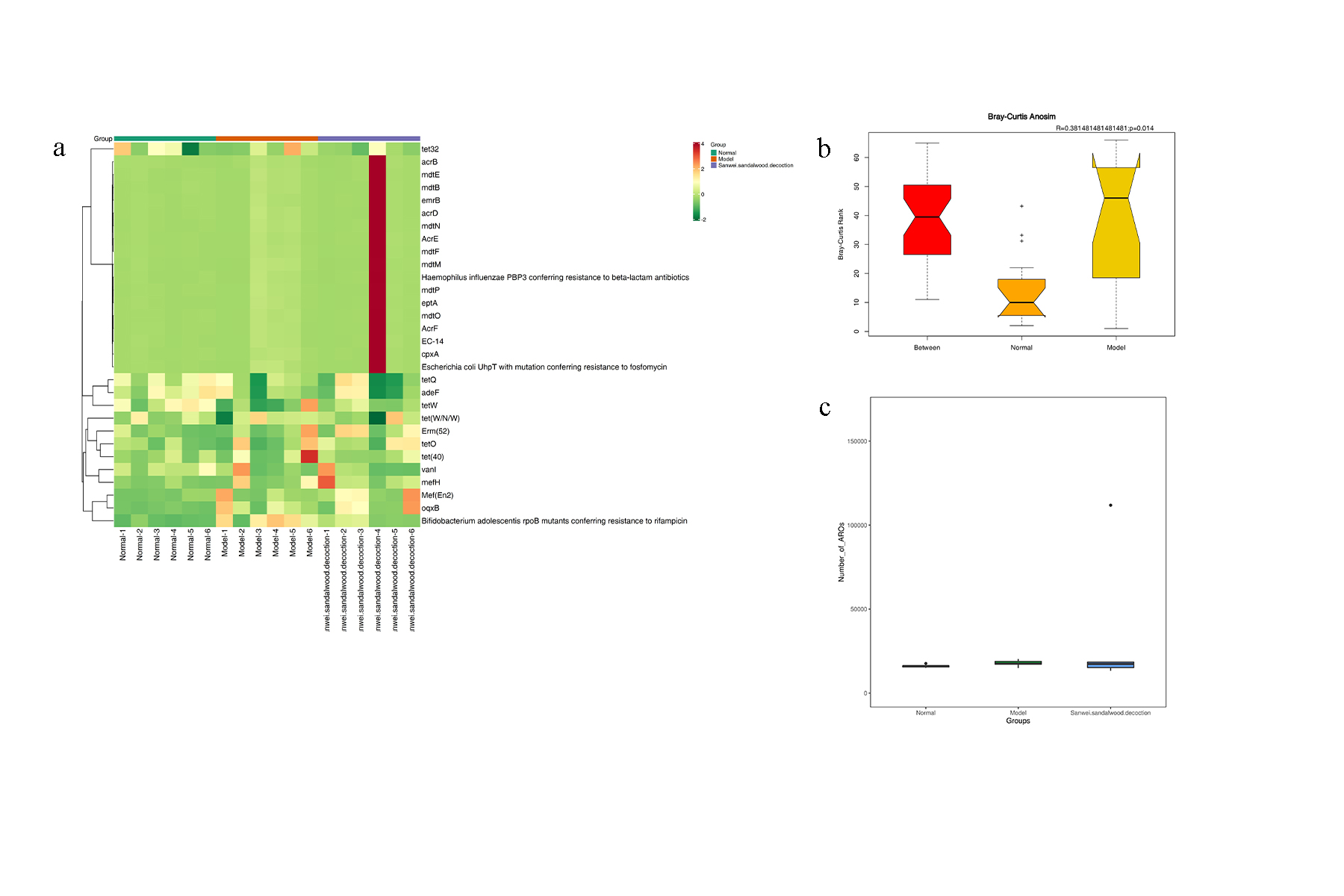

Supplement: Supplementary file 18 [file Image_3.JPEG]

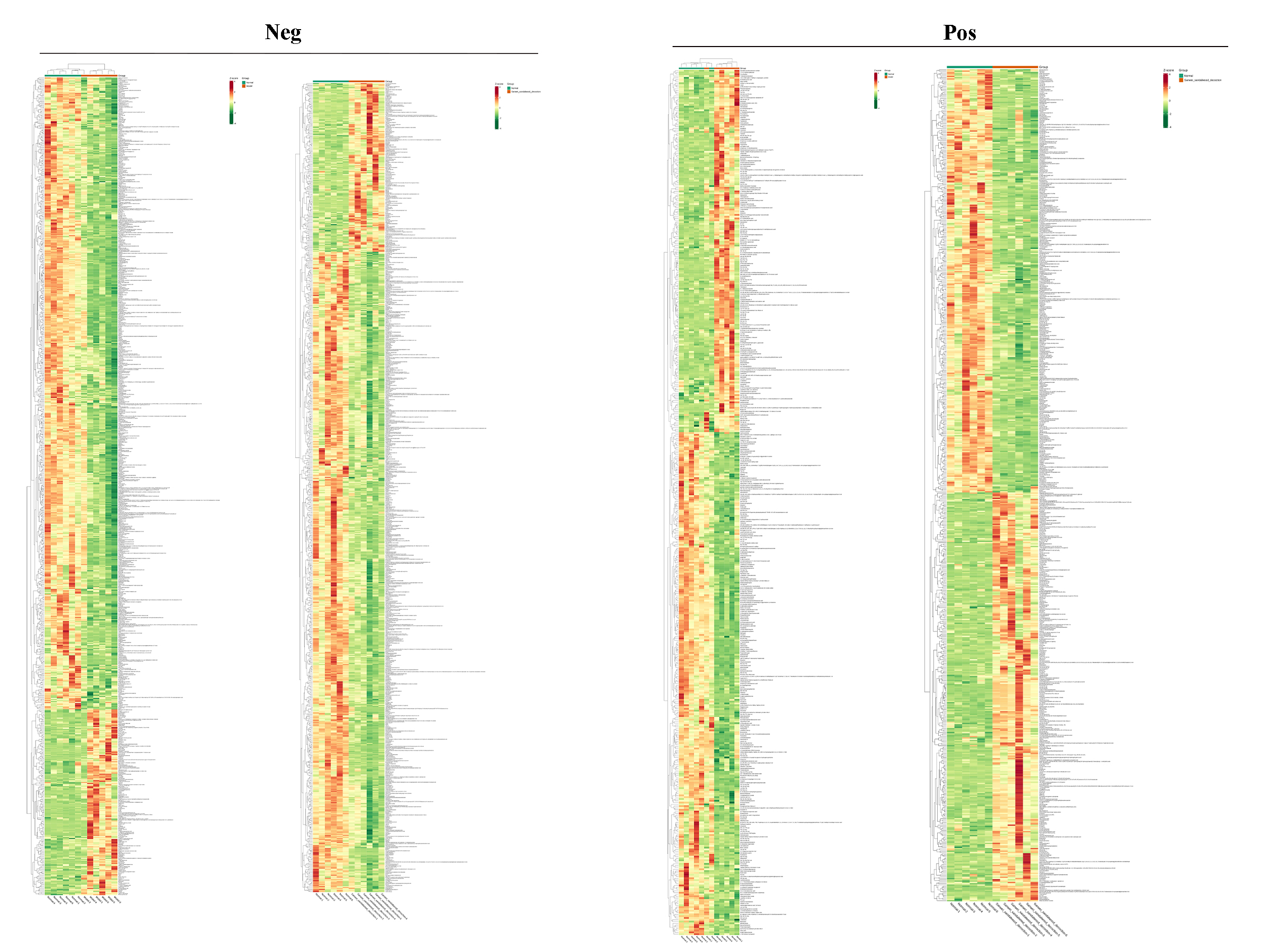

Supplement: Supplementary file 19 [file Image_4.JPEG]

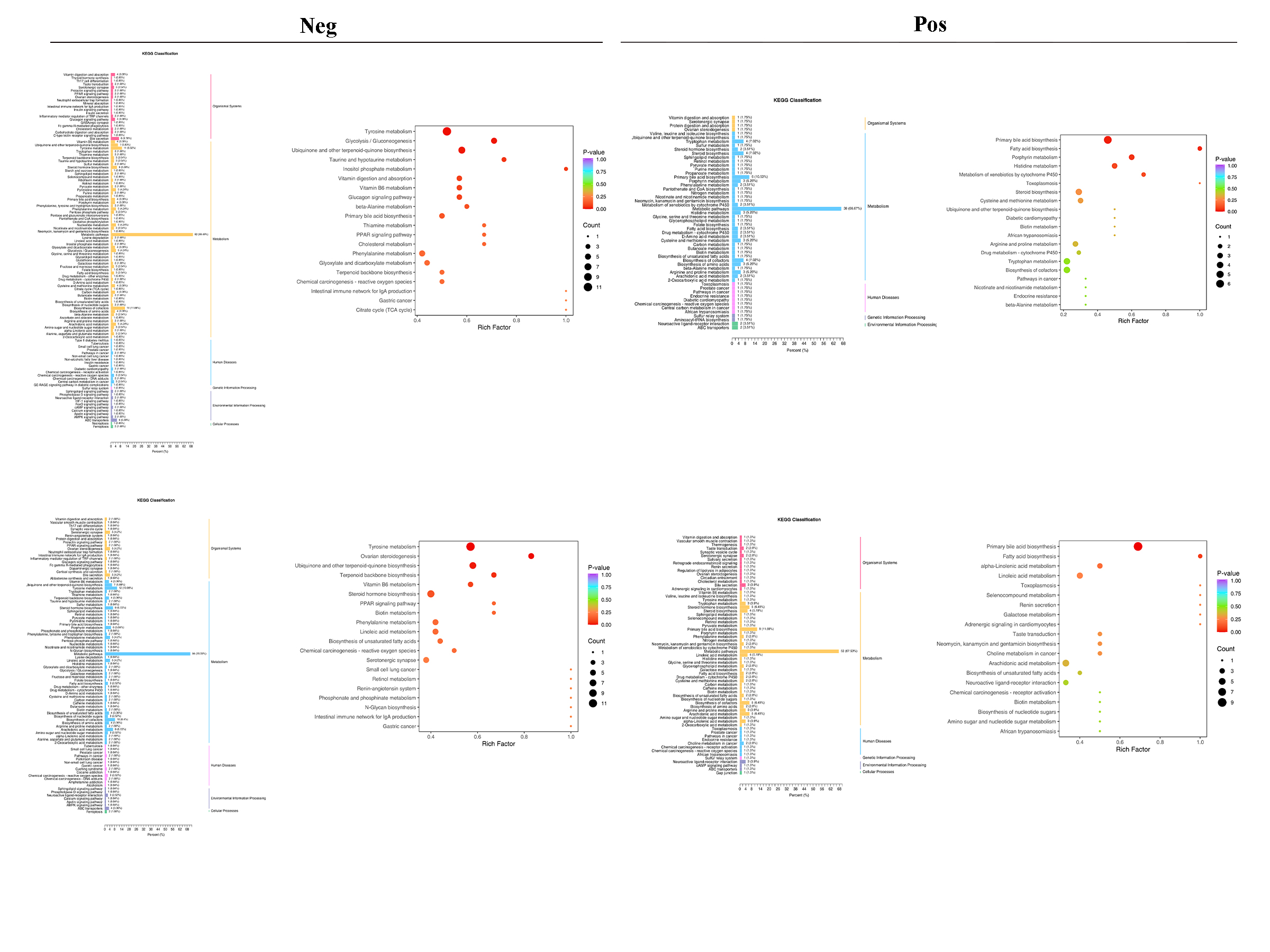

Supplement: Supplementary file 20 [file Image_5.JPEG]
